# Supplementary figures and images for: Pursuing the quest for better understanding the taxonomic distribution of the system of doubly uniparental inheritance of mtDNA
Source: PeerJ. 2016 Dec 13;4:e2760. doi: 10.7717/peerj.2760 (PMC5157197; doi:10.7717/peerj.2760)

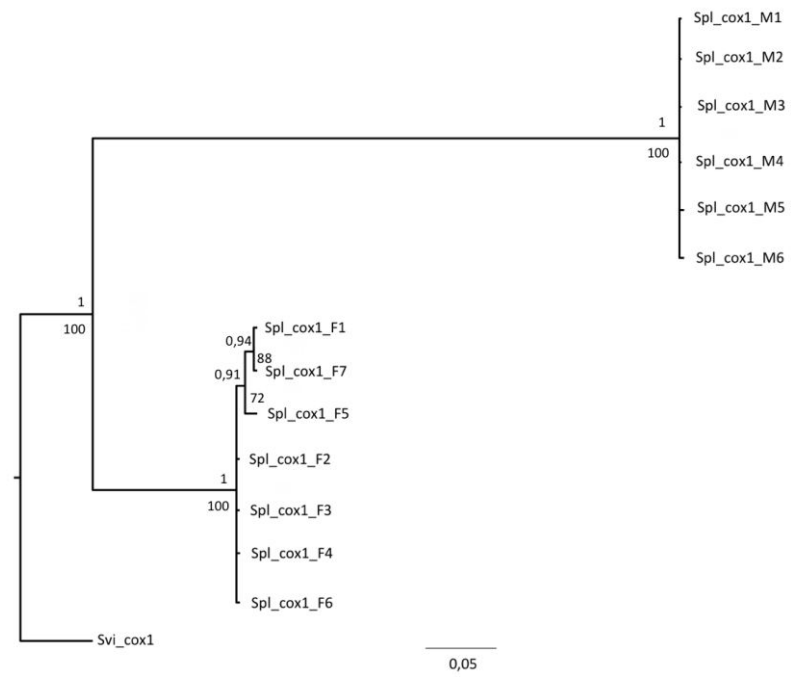

Supplement: Figure S1 — Numbers above the branches indicate the posterior probability determined from the Bayesian analyses and numbers below the branches refer to the bootstrap value estimated from the maximum-likelihood phylogeny. Soletellina virescens (Svi) was used as outgroup. [file peerj-04-2760-s001.pdf]

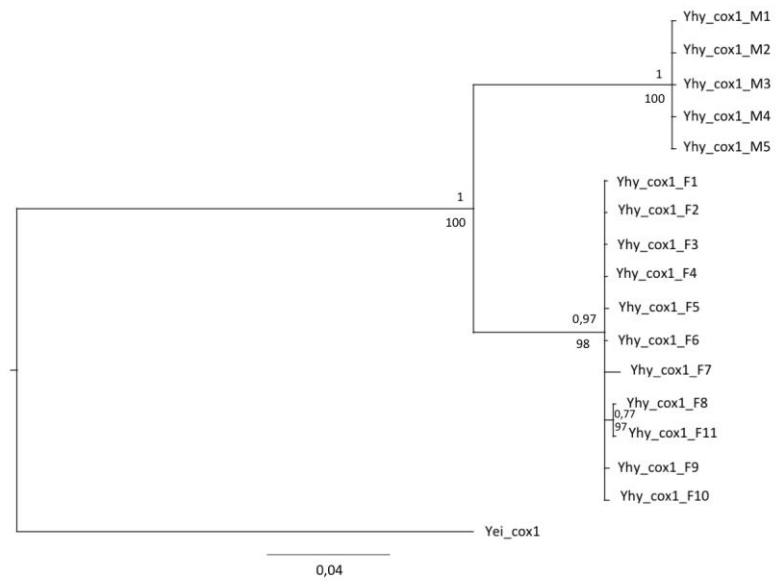

Supplement: Figure S2 — Numbers above the branches indicate the posterior probability determined from the Bayesian analyses and numbers below the branches refer to the bootstrap value estimated from the maximum-likelihood phylogeny. Yoldia eightsii (Yei) was used as outgroup. [file peerj-04-2760-s002.pdf]

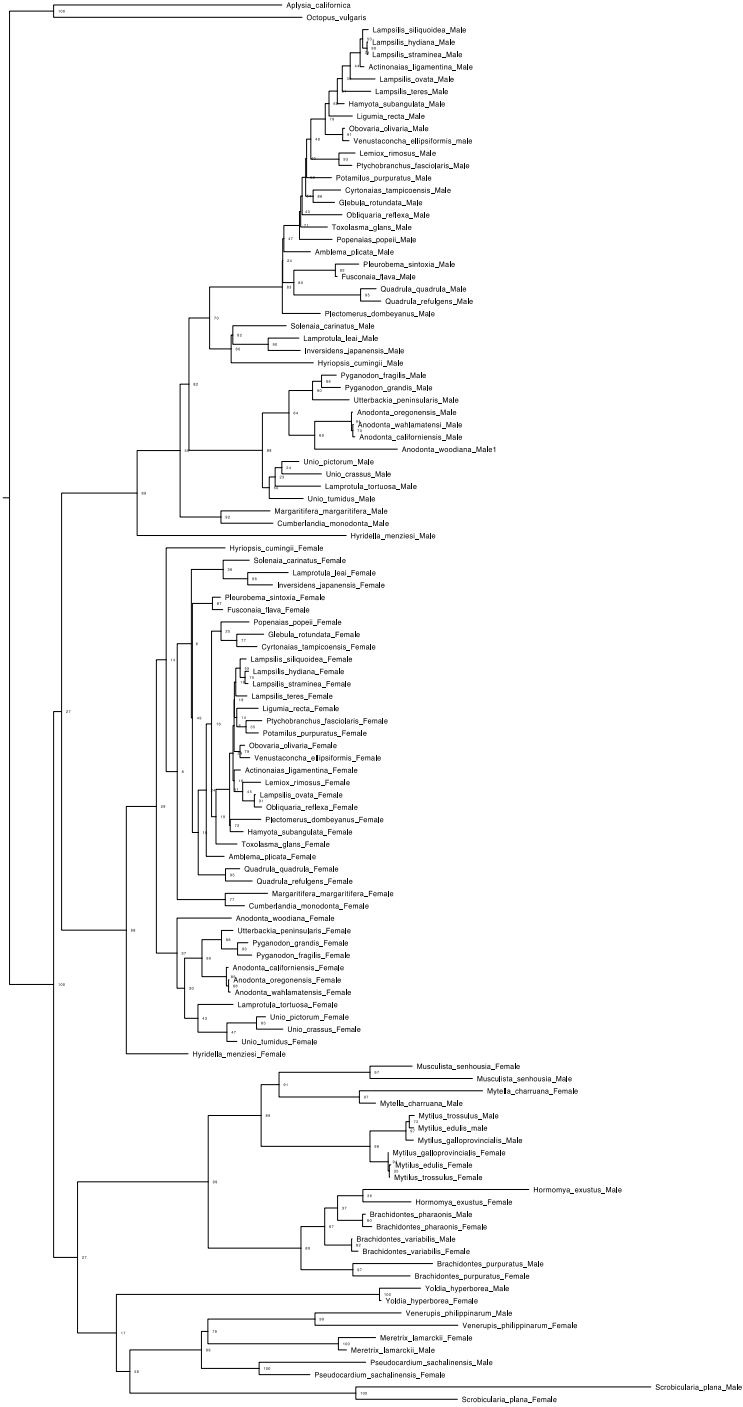

0.5

---

Outgroup

# Unionoida

Mytiloida

---

\* Veneroida

Supplement: Figure S3 — Based on an analysis using the GTR + I + G model. Bootstrap support appears next to each node. * : Nuculanoida. [file peerj-04-2760-s003.pdf]

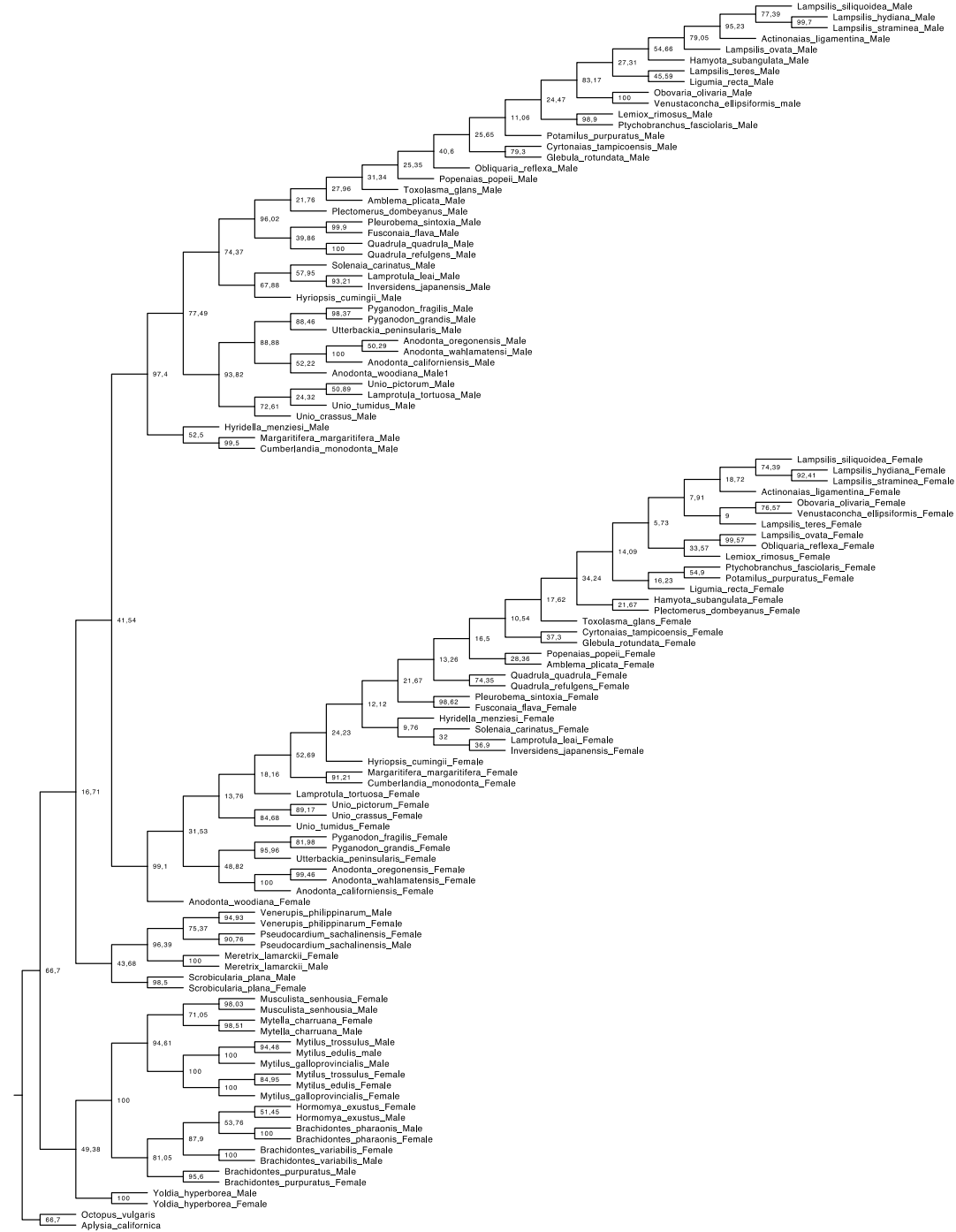

Supplement: Figure S4 — Bootstrap support appears next to each node. [file peerj-04-2760-s004.pdf]
