# Supplementary material for: Pursuing the quest for better understanding the taxonomic distribution of the system of doubly uniparental inheritance of mtDNA
Source: PeerJ. 2016 Dec 13;4:e2760. doi: 10.7717/peerj.2760 (PMC5157197; doi:10.7717/peerj.2760)
Supplement: Table S1 [file peerj-04-2760-s005.docx]

**Table S1.** List of the best fitting-models for the nucleotide phylogenetic analyses according to BIC value. The lower the BIC value, the better the model fits with the data. AIK, Akaike Information Criterion; BIC, Bayesian Information Criterion; INL, Natural log likelihood.

| Model | lNL | AIC | AICc | BIC |
| --- | --- | --- | --- | --- |
| TIM+I+G | -27348.31368 | 54712.62736 | 54712.8677607 | 54747.9087591 |
| TrN+I+G | -27355.2452 | 54724.4904 | 54724.6770667 | 54755.3616242 |
| GTR+I+G | -27347.43909 | 54714.87818 | 54715.2466892 | 54758.9799288 |
| K81uf+I+G | -27396.63346 | 54807.26692 | 54807.4535867 | 54838.1381442 |
| HKY+I+G | -27404.24135 | 54820.4827 | 54820.6224671 | 54846.9437493 |
| TVM+I+G | -27394.99722 | 54807.99444 | 54808.2954433 | 54847.6860139 |
| TIM+G | -27405.78878 | 54825.57756 | 54825.7642267 | 54856.4487842 |
| TrN+G | -27411.62272 | 54835.24544 | 54835.3852071 | 54861.7064893 |
| GTR+G | -27404.69273 | 54827.38546 | 54827.6864633 | 54867.0770339 |
| K81uf+G | -27446.11864 | 54904.23728 | 54904.3770471 | 54930.6983293 |
| HKY+G | -27453.17046 | 54916.34092 | 54916.4405878 | 54938.3917944 |
| TVM+G | -27444.83693 | 54905.67386 | 54905.9142607 | 54940.9552591 |
| SYM+I+G | -27909.2377 | 55832.4754 | 55832.6620667 | 55863.3466242 |
| SYM+G | -27971.29972 | 55954.59944 | 55954.7392071 | 55981.0604893 |
| TrNef+I+G | -28018.26796 | 56044.53592 | 56044.602255 | 56062.1766195 |
| TIMef+I+G | -28018.26073 | 56046.52146 | 56046.6211278 | 56068.5723344 |
| TrNef+G | -28081.77954 | 56169.55908 | 56169.5988151 | 56182.7896046 |
| TIMef+G | -28081.73505 | 56171.4701 | 56171.536435 | 56189.1107995 |
| TVMef+I+G | -28126.97001 | 56265.94002 | 56266.0797871 | 56292.4010693 |
| TVMef+G | -28179.47161 | 56368.94322 | 56369.0428878 | 56390.9940944 |
| K80+I+G | -28232.49492 | 56470.98984 | 56471.0295751 | 56484.2203646 |
| K81+I+G | -28232.47155 | 56472.9431 | 56473.009435 | 56490.5837995 |
| K80+G | -28286.95197 | 56577.90394 | 56577.9237747 | 56586.7242898 |
| K81+G | -28286.9476 | 56579.8952 | 56579.9349351 | 56593.1257246 |
| F81+I+G | -29494.62578 | 58999.25156 | 58999.3512278 | 59021.3024344 |
| F81+G | -29549.26104 | 59106.52208 | 59106.588415 | 59124.1627795 |
| JC+I+G | -29606.96222 | 59217.92444 | 59217.9442747 | 59226.7447898 |
| JC+G | -29657.59725 | 59317.1945 | 59317.2011007 | 59321.6046749 |
| SYM+I | -30205.50541 | 60423.01082 | 60423.1505871 | 60449.4718693 |
| GTR+I | -30244.99657 | 60507.99314 | 60508.2941433 | 60547.6847139 |
| TIM+I | -30346.90562 | 60707.81124 | 60707.9979067 | 60738.6824642 |
| TrN+I | -30350.58853 | 60713.17706 | 60713.3168271 | 60739.6381093 |
| TVM+I | -30436.74696 | 60889.49392 | 60889.7343207 | 60924.7753191 |
| TrNef+I | -30494.24549 | 60994.49098 | 60994.5307151 | 61007.7215046 |
| TIMef+I | -30494.16869 | 60996.33738 | 60996.403715 | 61013.9780795 |
| TVMef+I | -30516.54577 | 61043.09154 | 61043.1912078 | 61065.1424144 |
| K81uf+I | -30543.65254 | 61099.30508 | 61099.4448471 | 61125.7661293 |
| HKY+I | -30548.21657 | 61106.43314 | 61106.5328078 | 61128.4840144 |
| K80+I | -30799.77759 | 61603.55518 | 61603.5750147 | 61612.3755298 |
| K81+I | -30799.56789 | 61605.13578 | 61605.1755151 | 61618.3663046 |
| JC+I | -32005.11367 | 64012.22734 | 64012.2339407 | 64016.6375149 |
| F81+I | -32145.88992 | 64299.77984 | 64299.846175 | 64317.4205395 |
| SYM | -32172.57059 | 64355.14118 | 64355.2408478 | 64377.1920544 |
| GTR | -32309.9915 | 64635.983 | 64636.2234007 | 64671.2643991 |
| TIM | -32419.87145 | 64851.7429 | 64851.8826671 | 64878.2039493 |
| TrN | -32427.1851 | 64864.3702 | 64864.4698678 | 64886.4210744 |
| TVMef | -32467.34254 | 64942.68508 | 64942.751415 | 64960.3257795 |
| TrNef | -32491.4662 | 64986.9324 | 64986.9522347 | 64995.7527498 |
| TIMef | -32490.40571 | 64986.81142 | 64986.8511551 | 65000.0419446 |
| TVM | -32524.97601 | 65063.95202 | 65064.1386867 | 65094.8232442 |
| K81uf | -32641.29106 | 65292.58212 | 65292.6817878 | 65314.6329944 |
| HKY | -32649.67244 | 65307.34488 | 65307.411215 | 65324.9855795 |
| K80 | -32786.48039 | 65574.96078 | 65574.9673807 | 65579.3709549 |
| K81 | -32785.15545 | 65574.3109 | 65574.3307347 | 65583.1312498 |
| JC | -33919.74979 | 67839.49958 | 67839.49958 | 67839.49958 |
| F81 | -34124.66017 | 68255.32034 | 68255.3600751 | 68268.5508646 |
